# Sox11 is enriched in myogenic progenitors but dispensable for development and regeneration of the skeletal muscle

**DOI:** 10.1186/s13395-023-00324-0

**Published:** 2023-09-13

**Authors:** Stephanie N. Oprescu, Nick Baumann, Xiyue Chen, Qiang Sun, Yu Zhao, Feng Yue, Huating Wang, Shihuan Kuang

**Affiliations:** 1 Department of Biological Sciences, Purdue University, West Lafayette, IN 47907 USA; 2 Department of Animal Sciences, Purdue University, West Lafayette, IN 47907 USA; 3 Department of Orthopedics and Traumatology, Li Ka Shing Institute of Health Sciences, The Chinese University of Hong Kong; Center for Neuromusculoskeletal Restorative Medicine, Hong Kong Science Park, Hong Kong, China; 4 Center for Cancer Research, Purdue University, West Lafayette, IN 47907 USA

**Keywords:** Aging, Differentiation, Satellite cells, Single-cell RNA-sequencing (scRNA-seq), SRY-box transcription factor, Stem cells

## Abstract

Transcription factors (TFs) play key roles in regulating differentiation and function of stem cells, including muscle satellite cells (MuSCs), a resident stem cell population responsible for postnatal regeneration of the skeletal muscle. Sox11 belongs to the Sry-related HMG-box (SOX) family of TFs that play diverse roles in stem cell behavior and tissue specification. Analysis of single-cell RNA-sequencing (scRNA-seq) datasets identify a specific enrichment of *Sox11* mRNA in differentiating but not quiescent MuSCs. Consistent with the scRNA-seq data, *Sox11* levels increase during differentiation of murine primary myoblasts in vitro. scRNA-seq data comparing muscle regeneration in young and old mice further demonstrate that *Sox11* expression is reduced in aged MuSCs. Age-related decline of *Sox11* expression is associated with reduced chromatin contacts within the topologically associating domains. Unexpectedly, Myod1^Cre^-driven deletion of *Sox11* in embryonic myoblasts has no effects on muscle development and growth, resulting in apparently healthy muscles that regenerate normally. Pax7^CreER^- or Rosa26^CreER^- driven (MuSC-specific or global) deletion of *Sox11* in adult mice similarly has no effects on MuSC differentiation or muscle regeneration. These results identify Sox11 as a novel myogenic differentiation marker with reduced expression in quiescent and aged MuSCs, but the specific function of Sox11 in myogenesis remains to be elucidated.

**Supplementary Information:**

The online version contains supplementary material available at 10.1186/s13395-023-00324-0.

## Background

Skeletal muscle makes up nearly 40% of the total body mass and is critical for balance, movement, and maintaining quality of life [1]. As a surface tissue, the skeletal muscle is prone to various injuries. Mammalian skeletal muscles harbor a resident population of adult stem cells, known as muscle satellite cells (MuSCs), which in response to external stimuli, such as an injury, activate, proliferate, and differentiate to repair the injured muscle. While various infiltrating and resident cells are necessary for clearing debris, remodeling the extracellular matrix, and modulating the regenerative environment, MuSCs critically contribute to the repair by fusing together to generate new fibers to restore muscle function [2]. To fully repair the muscle, MuSCs must first exit their quiescent state, proliferate to expand the pool, and then either commit to the myogenic program and differentiate or maintain their stem-like state and self-renew to replenish the adult MuSC pool. This balance of self-renewal and differentiation is critical for muscle function, as dysregulation can lead to exhaustion of the MuSC pool and impair subsequent regenerative capacity under pathological conditions [3–5]. While external signals in the environment impact the MuSC response, these signals must be integrated within the cell to ultimately affect cell identity and cell fate decisions [6]. How a cell responds to various cues is mediated by its transcriptional state, underscoring the importance of key transcriptional regulators in cell identity and cell states.

Muscle stem cell activation, proliferation, and differentiation during muscle repair recapitulates various aspects of the myogenic developmental process, and the major transcriptional regulators of this process are well understood [6–13]. The best-known transcription factors are Pax3/Pax7 and the myogenic regulatory factors (MRFs, which include Myf5, Myf6, Myod1, and myogenin) [6–9, 14–18]. The expression of these factors is MuSC-state specific, with Pax3/Pax7 primarily expressed in quiescent and proliferating MuSCs, while the MRFs are differentially expressed along the myogenic lineage [19]. Cells expressing Pax7 are generally refractory to differentiation cues and will maintain their self-renewing state by controlling transcription of quiescence-related genes, which is mediated by the transcriptional state of MuSCs and is influenced by external environmental signals [20, 21]. Thus, transcriptional states and integration of external environmental signals are relatively complex. Notch signaling is one of the best characterized pathways that play a critical role in maintaining the balance of MuSC quiescence and self-renewal [20], through regulation of Hes/Hey family transcriptional factors. During development, the Notch ligand Delta-like 1 (Dll1) regulates myogenic differentiation and maintenance of progenitors, as high expression of the Notch intracellular domain (NICD) supports their proliferation [22, 23]. Conversely, in adult MuSCs, high NICD expression maintains their quiescence, in part by targeting the expression of niche-related collagen genes and miR-708, which limits migration and further reinforces quiescence through niche interactions [24–26]. As MuSCs proliferate, high expression NICD supports *Pax7* expression to promote the self-renewal of MuSCs [27, 28]. Interestingly, changes in the expression of Notch ligands and reduced p53 expression have been linked to age-related functional decline of MuSCs [29, 30]. In addition to the critical role of Notch signaling, Wnt signaling has also been shown to mediate MuSC function, also through altering a transcriptional program mediated by β-catenin. For example, Wnt1 + cells preferentially activate *Myf5*, while cells expressing Wnt7a preferentially activate *Myod* [31, 32]. In response to muscle injury, canonical Wnt/β-catenin signaling promotes commitment of myoblasts by regulating the expression of follistatin [33]. Age-related increase in Wnt signaling was also found to mediate the conversion of MuSCs to a more fibrogenic-like state, leading to impaired regeneration [34]. Overall, these findings exemplify the role of transcriptional factors in mediating external signals to regulate MuSC state and function.

**Fig. 1:**
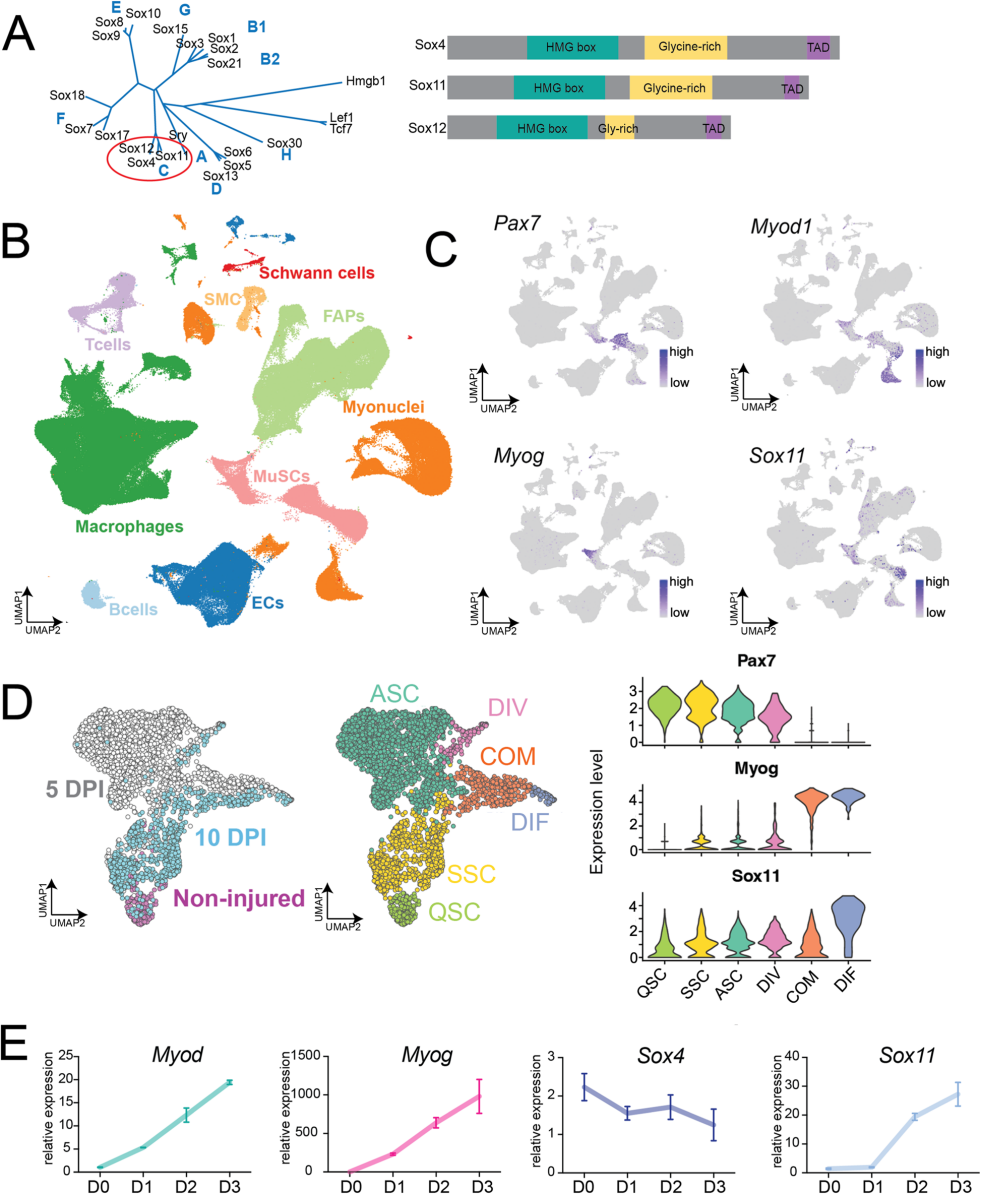
Single-cell RNA-sequencing identified *Sox11* expression in differentiating muscle stem cells. **A** Phylogenetic neighbor-joining tree for the high-mobility group (HMG) domain containing Sox factors highlighting the SoxC subfamily and their domain phylogenetic similarity for mammals [44]*.* **B** UMAP plot of McKellar et al., aggregated dataset (GEO accession: GSE162172), broadly classified by cell type. **C** UMAP-based gene expression plots from McKellar dataset for *Pax7*, *Myod1*, *Myog*, and *Sox11*. **D** UMAP projection of scRNA-seq of MuSCs sorted from non-injured, 5 and 10 DPI muscle, colored by timepoint and cluster identity (left panel) (GEO accession: GSE150366). Violin plot of *Pax7*, *Myog*, and *Sox11* based on scRNA-seq dataset to highlight expression in different MuSCs clusters. **E** qRT-PCR on RNA isolated from proliferating (D0) myoblasts and myoblasts induced to differentiate for 1, 2, and 3 days to detect the changes in expression of *Myod1*, *myogenin*, *Sox11*, and *Sox4*

To identify factors that regulate transcriptional states that may regulate or sensitize MuSC fate decisions, we employed published and newly generated single-cell RNA-sequencing data to identify *Sox11* as a transcriptional factor enriched in differentiating MuSCs. The SOX protein family is best known for their role in development, embryonic stem cells, tissue specification, and sex determination [35, 36]. Among the 20 SOX proteins, Sox11 is a member of the SoxC subfamily, which also includes Sox4 and Sox12, all of which are widely expressed during embryogenesis and are developmentally required (Fig. 1A) [36, 37]. Although Sox4 and Sox11 exhibit some redundancy, they are both critical for cell and embryo survival. *Sox11 − / − *mice are born viable but do not survive past 24 h due to impaired organogenesis such as under-mineralized bones and heart malformations [38–40]. Sox11 is broadly required for survival of neural and mesenchymal progenitors and mediates the proliferation of neural progenitors in the central nervous system while promoting precursor differentiation in the peripheral nervous system [7, 38, 41–43]. These findings underscore the complex, cell and tissue-type specific regulatory role for Sox11 during development.

The role of Sox11 during adulthood has also been investigated in several disease and regeneration settings. High expression of *Sox11* in various cancers is generally correlated with poor prognosis as it appears to support the epithelial-to-mesenchymal transition of cancer cells [45]. Studies on the role of Sox11 in tumorigenesis indicate that Sox11 regulates genes involved in Wnt signaling and the Notch pathway, both of which are imperative for MuSC function [27, 28, 33, 34, 46–49]. Sox11 also regulates *Tead2* expression to support the survival and proliferation of osteoblasts and mesenchymal cells while regulating *Osterix* and *Runx2* to promote osteoblast and mesenchymal cell differentiation [50]. Interestingly, Sox11 was also shown to mediate sensory nerve regeneration, as knockdown of *Sox11* RNA inhibited regeneration in vivo in adult mice [41, 51]. Additionally, Sox11 (and another member of the SoxC subfamily, Sox4) was found to reactivate embryonic developmental programs to support skin wound repair by inhibiting premature differentiation [52]. However, the expression and function of Sox11 in MuSCs and muscle regeneration have not been investigated.

Several members of the Sox family of transcription factors have been reported to play a role in myogenesis. For example, Sox6 functions to repress slow-fiber type gene expression, while Sox8 negatively regulates MuSC differentiation [53–55]. Additionally, a previous study identified another member of the SoxC subfamily, Sox4, is important for differentiation of the muscle cell line, C2C12, by targeting the *Cald1* promoter [56]. However, unlike *Sox11*, we found that *Sox4* expression is not restricted to MuSCs and does not increase during primary myoblast differentiation, consistent with a previous study that detected increased expression of *Sox11* during myoblast differentiation [53].

While several of the Sox family members are known to play a role in skeletal muscle function, the role for Sox11 has not been described. However, Sox11 has been identified as a regulator of WNT signaling pathway components. For example, Sox11 represses WNT signaling in mantel cell lymphoma [57] and was shown to regulate the transcription of β-catenin in rat mesenchymal stem cells, which is consistent with previous reports that the SoxC family synergize with WNT signaling components to stabilize signaling events during skeletogenesis [58, 59]. While the role for WNT signaling in skeletal muscle development is well defined, the requirement for WNT signaling for MuSC function in response to muscle injury is somewhat unclear. For example, Wnt4a expression increased during MuSC differentiation, and addition of this ligand to C2C12s in vitro led to increased fusion and differentiation [60]. In vivo, Wnt ligands promoted muscle repair by antagonizing Notch signaling to support the differentiation of MuSCs [34]. Nonetheless, while Wnt signaling is activated during muscle repair, the necessity of Wnt signaling remains unclear. For example, an early report indicated that the loss of β-catenin minimally impacts muscle regeneration, while later reports suggest that the Wnt/β-catenin signaling pathway is necessary for MuSC differentiation [49, 61]. In addition to the canonical role of Wnt signaling which is primarily mediated through β-catenin, noncanonical Wnt signaling through Wnt7a regulates MuSC self-renewal and in mature fibers promotes hypertrophy through Akt/mTOR signaling [62, 63]. These studies underscore, necessitating a genetic approach to understand the potential novel regulators of the WNT pathway as well as other pathways that mediate MuSC function.

Given the enrichment of Sox11 in myogenic cells and the broad regulatory role of Sox11 in other non-muscle tissues, we hypothesized that Sox11 may play a unique role in myogenesis and could potentially help us understand the regulation of WNT signaling in muscle stem cell function and repair. In the present study, we utilized a handful of scRNA-seq datasets from acutely injured skeletal muscle to identify an enriched *Sox11* expression in differentiating MuSCs. We subsequently investigated the requirement of Sox11 muscle progenitor function using various conditional knockout mouse models. While these data suggest that Sox11 is dispensable for normal MuSC function under the evaluated conditions, it adds to our understanding of the potential redundancy of the SOX family members in myogenesis.

## Methods

### Muscle injury and sample processing for single-cell RNA-sequencing

Hindlimb muscle of 3 *Pax7*^nGFP/+^ male mice at 3–4 months of age (young) and > 20 months of age (old) was injured via intramuscular injection of 50-µL 10-µM cardiotoxin.

Hindlimb muscles were dissected at 7 DPI, digested to release mononuclear cells, and sorted via fluorescence-activated cell sorting to select for live, single cells as previously described [64, 65].

### Single-cell RNA-sequencing

scRNA-sequencing was performed using the 10 × Genomics 3′ v2 kit, following their protocol targeting recovery of 10,000 cells. Libraries were constructed per the manufacturer’s instructions, sequenced on Illumina’s NovaSeq platform. Reads were aligned to the mouse genome mm10/Grcm38 using the CellRanger 2.1.0 software, and additional analysis was performed in R.

### Quality control, dimensionality reduction, and visualization

Seurat 3.1.0 in R was used to analyze CellRanger output following a broadly similar pipeline as previously described [66]. Both young and old samples were merged and filtered for cells with less than 15% reads mapping to mitochondrial genes, gene counts less than 6000 per cell, and no more than 60,000 reads. Both Seurat’s SCTransform function and log-transformation methods were used to normalize and scale the data and subsequently compare results, which yielded similar outcomes (data not shown) [67]. Dimensionality reduction was performed through principal component analysis (PCA), and top principal components were selected by evaluating elbow plots. Clustering and UMAP embedding parameters were based on the top 10 PCs and embedded in 2 dimensions for visualization. FindAllMarkers() function was used to identify gene enriched in each cluster, which were used to manually label cell types. All genes considered for cell-type classification had a *P*-value of less than 0.0001 using a Mann–Whitney Wilcoxon test. To perform the sub-clustering, we used Seurat’s subset function to extract the cell types of interest (MuSCs), we extracted the raw RNA counts for each assayed cell type to subset, rescaled the data using the SCTransform function, and performed dimensionality reduction, clustering, and UMAP visualization. We then compared gene expression based on cell sample (i.e., age). Additional scRNA-sequencing data is based on previously analyzed and published datasets [68, 69].

### Gene expression analysis of RNA-seq data

The raw reads of total RNA-seq were processed following the procedures described in previous publication. Briefly, the adapter and low-quality sequences were trimmed, and the reads shorter than 50 bp were discarded. The clean reads were mapped to mouse genome (mm9) with Bowtie2 (V. 2.1.1). Cuff links (V 2.2.1) were then used to estimate gene expression level in Fragments Per Kilobase per Million (FPKM). Genes were annotated as differentially expressed if the change of expression level is greater than 2-folds between two stages/conditions.

### ChIP-seq data analysis

Raw ChIP-seq reads were processed as previously described [70]. Briefly, the adapter and low-quality sequences were trimmed from 3′ to 5′ ends by Trimmomatic (V 0.36), and the reads shorter than 36 bp were discarded. Subsequently, the preprocessed reads were aligned to the mouse genome (mm9) using Bowtie2 (v2.3.3.1). The duplicate reads were removed by Picard (http://broadinstitute.github.io/picard). Peaks were then identified by MACS2 (V 2.2.4) with *q*-value equal to 0.01 by using the IgG control sample as background.

### In situ Hi-C data processing

The in-situ Hi-C data was processed with HiC-Pro (v2.10.0) [71]. First, adaptor sequences and poor-quality reads were removed using Trimmomatic (ILLUMINACLIP: TruSeq3-PE-2.fa:2:30:10; SLIDINGWINDOW: 4:15; MINLEN:50). The filtered reads were then aligned to reference genome (mm9). All aligned reads were then merged together and assigned to restriction fragment, while low-quality (*MAPQ* < 30) or multiple alignment reads were discarded. Invalid fragments including unpaired fragments (singleton), juxtaposed fragments (re-legation pairs), un-ligated fragments (dangling end), self-circularized fragments (self-cycle), and PCR duplicates were removed from each biological replicate. The remaining validate pairs from all replicates of each stage were then merged, followed by read depth normalization using HOMER (http://homer.ucsd.edu/homer/interactions/HiCpca.html) and matrix balancing using iterative correction and eigenvector decomposition (ICE) normalization to obtain comparable interaction matrix between different stages.

### Identification and analysis of TADs

Normalized contact matrix at 10-kb resolution of each time point was used for TAD identification using TopDom (v. 0.0.2) [72]. In brief, for each 10-kb bin across the genome, a signal of the average interaction frequency of all pairs of genome regions within a distinct window centered on this bin was calculated; thus, TAD boundary was identified with local minimal signal within certain window. The insulation score of the identified TAD border was also defined as previously described, which used the local maximum on the outside of TAD to minus the local minimum on the inside of TAD of each boundary bin.

### In silico knockout analysis

Functional analysis of *Sox11* was conducted using the R package of *scTenifoldKnk* [73]. A single-cell gene regulatory network (scGRN) was conducted on muscle satellite cells from the control samples in our previously published scRNA-seq dataset ([69]; GSE150366). Then, the expression of *Sox11* was set to zero from the constructed scGRN to build their own corresponding “pseudo-knockout” scGRN. Perturbed genes by this virtual knockout were quantified by comparison of the “pseudo-knockout” scGRN to the original scGRN. Those significantly affected genes were used for functional enrichment analysis (GO and KEGG) to show changes in biological processes caused by in silico knockout.

### Animals

Animals used in this study were as follows: Pax7^CreERT2^ (no. 017763), Rosa26^CreER^ (no. 008463), and Myod1^Cre^ (#014140) were purchased from Jackson Laboratory as the respective stock number. Sox11^flox/flox^ were a gift from Dr. Veronique Lefebvre (Children’s Hospital of Philadelphia). Mouse genotypes were evaluated by genomic DNA isolation from the ear and determined via polymerase chain reaction (PCR). For mice purchased from Jackson Laboratory, primers and protocols published with each. For Sox11, genotyping for the floxed allele was determined via PCR as previously described [74]. DNA Recombination PCR was performed using primers specific for the recombined allele on DNA isolated from whole muscle or myoblasts treated with MeOH or 4OH, described in the section titled "genomic DNA isolation and recombination PCR" below [74]. A 3–6-month-old mice were used and were always age and litter matched. Littermate controls included both Cre-positive heterozygous floxed mice and Cre-negative mice. No sex-specific differences were observed. All procedures and mice were approved and housed according to the Purdue Animal Care and Use Committee standards.

### Tamoxifen

For *Pax7*^CreER^ and *Rosa26*^CreER^ mice, tamoxifen was in to induce recombination of the floxed allele. Tamoxifen (100 mg/mL) was administered via intraperitoneal injection of 100 µL per 10 g of body weight. Mice were injected with tamoxifen 4 consecutive days in a row > 1 week prior to analysis and subsequent the day prior to injury injected with tamoxifen again to ensure recombination of the floxed allele (to total 5 injections).

### Muscle injury

Skeletal muscle injury was induced via tibialis anterior intramuscular of 50 µL 10-µM cardiotoxin or 50 µL 1.2% weight/volume of barium chloride (BaCl_2_) using 27-gauge needle. Mice were first anesthetized using a ketamine/xylazine cocktail, and intramuscular injection of the respective toxin was induced using a 27-gauge needle. Muscle samples were collected at the respective timepoints after injury and analyzed as described below. The contralateral muscle always served as a non-injured control.

### Cross sectioning muscle analysis

Cardiotoxin or BaCl_2_-injured muscle and the respective contralateral non-injured controls were collected at the respective timepoints after injury, weighed and embedded in O.C.T. (Fisher, 4585), and flash-frozen to preserve muscle tissue. Samples were cross-sectioned at 10-µm thickness using a Leica CM1850 Cryostat set at − 20 °C. Muscle sections were placed on a Tek-Slide (IMEB Inc., cat. no. Y-9253), processed for hematoxylin and eosin staining, immunofluorescence, or stored at − 80 °C.

### Hematoxylin and eosin staining

Muscle cross-sections were first placed in hematoxylin for 15 min, rinsed with gently running water for 1–2 min, placed in eosin for 1 min, placed in ethanol to dehydrate samples (70%, 95%, 100%, 1 min each) and then xylene for 2 min, and covered using Permount and a glass cover slip.

### Immunofluorescence staining

For slides, samples were surrounded by tissue blocker pen (for muscle fibers and myoblasts, samples were processed in 24- or 48-well plates) and fixed with 4% paraformaldehyde (PFA) for 10 min at room temperature. Samples were washed 3 times with 1 × PBS (pH 7.5, 5 min per wash), incubated with 1 × glycine (0.375 g/50 mL dissolved in 1 × PBS) for 10 min, and washed again 3 times with 1 × PBS. Samples were blocked for 1 h at room temperature in blocking buffer (5% goat serum, 2% bovine serum album, 0.1% Triton X-100, 0.1% sodium azide prepared in 1 × PBS). Primary antibodies were diluted in blocking buffer, and samples were stained with the primary antibody overnight at 4 °C. Samples were calibrated to room temperature and washed with 1 × PBST (1% Tween 20 in 1 × PBS) 3 times at room temperature (5-min incubations). Samples were incubated with secondary antibodies and DAPI for 1 h at room temperature, washed 3 × with 1 × PBST, and a drop of Permount was added and a coverslip placed on top to preserve fluorescence.

Primary antibodies.

**Table:** 

Name	Catalog no	Host
Anti-Pax7	DSHB cat. no. RRID:AB_2299243	msIgG1
Anti-dystrophin	Abcam cat. no. ab15277, RRID:AB_301813	Rabbit
Anti-myogenin	DSHB cat. no. F5D, RRID:AB_2146602	msIgG1
Anti-type IIA	DSHB cat. no. SC-71	mIgG1
Anti-type IIB	DSHB cat. no. BF-F3	msIgM
Anti-type I	DSHB cat. no. BA-D5	msIgG2b
Anti-MF20 (MyHC)	DSHB cat. no. MF 20, RRID:AB_2147781	
Anti-Ki67	Abcam cat. no. ab15580, RRID:AB_443209	Rabbit
Anti-Myod	Santa Cruz Biotechnology cat. no. sc-377460, RRID:AB_2813894	msIgG2b
Anti-eMYHC	DSHB cat. no. F1.652, RRID:AB_528358	msIgG1
Anti-Laminin	Sigma cat. no. L9393, RRID:AB_477163	Rabbit

Secondary antibodies.

**Table:** 

Name	Catalog no
Alexa 568 goat anti-mouse IgG1	Invitrogen cat. no. A-21124, RRID:AB_2535766
Alexa 488 goat anti-mouse IgG1	Invitrogen cat. no. A-21121, RRID:AB_2535764
Alexa 647 goat anti-mouse IgG2b	Invitrogen cat. no. A-21242, RRID:AB_2535811
Alexa 488 goat anti-rabbit IgG	Invitrogen cat. no. A-11034, RRID:AB_2576217
Alexa 647 goat anti-rabbit IgG	Invitrogen cat. no. A-21244, RRID:AB_2535812

### Imaging

All H&E and immunofluorescence samples were captured using a 20 × or 10 × objective on the Leica DM6000B microscope. For each timepoint and pair, at least 4 images/genotypes were collected for analysis. Entire muscle images used for MuscleJ fiber-type analysis were captured using the 10 × objective on the Echo Revolution.

### Fluorescence-activated cell sorting

Muscle stem cells were purified based on a previously established protocol. Briefly, mice were injected with tamoxifen for 4 consecutive days, the hindlimb skeletal muscle injured via administration of BaCl, and samples were processed at 12 days after acute injury.

Skeletal muscles from each mouse were dissected, and samples were prepared for fluorescence activated cell sorting (FACS) as previously described [65]. After dissection of the skeletal muscle, muscle was rinsed with 1 × PBS, cut into pieces, and digested in wash media (F-10 + 10% horse serum + 1 × pen/strep) with 2.5 mg/mL collagenase type II (Worthington Biochemical Corporation, cat. no. L5004177) for 1 h at 37 °C. The sample was neutralized with 40-mL wash media, centrifuged at 500 × g for 5 min, and 40 mL of supernatant was removed leaving 8 mL of wash media. To this, 1 mL of 1000 U/mL stock collagenase type II and 1 mL of 11 U/mL dispase (Roche, cat. no. 04942078001) were added, and the samples were incubated at 37 °C for 30 min. Digested tissue was run through a 20-gauge needle to further dissociate cells, neutralized with 40-mL wash media and spun down at 500 × g for 5 min at room temperature (~ 22 °C). All but 10 mL of supernatant was removed, and cells were then resuspended, run through a 40-µm filter, diluted with 40-mL wash media, and subsequently spun down at 500 × g for 5 min at room temperature. Samples were resuspended in wash medium and incubated with FACS antibodies against CD31 (BD Biosciences, cat. no. 553373, RRID:AB_394819), Cd45 (eBioscience, cat. no. 12–0451-82, RRID:AB_465668), SCA1 (BioLegend, cat. no. 122520, RRID:AB_2143237), and VCAM1 (BioLegend, cat. no. 105718, RRID:AB_1877141) at a concentration of 1:500 and incubated at 4 °C for 20 min. Cells were then briefly spun down, washed once with 1 × PBS and resuspended in wash medium for fluorescence-activated cell sorting (FACS) to collect CD31-/Cd45-/SCA1-/VCAM1 + MuSCs.

### Genomic DNA isolation and recombination PCR

Approximately, 100 mg of fresh tissue was finely chopped in 500 µl of lysis buffer containing 0.05-M EDTA and 10-mM Tris–HCl at pH 8.0. Subsequently, 50 µl of 10% SDS and 2 µl of 20 mg/ml proteinase K were added and thoroughly mixed. The samples were then incubated at 65 °C for a minimum of 5 h or overnight. Afterward, 200 µl of 6-M NaCl (prepared in saturated NaCl solution) was added to each sample, followed by vigorous vortexing for 30 s at maximum speed. The tubes were then centrifuged at 10,000 g for 30 min, and the resulting supernatant was carefully transferred to fresh tubes. To each sample, an equal volume of isopropanol was added, thoroughly mixed, and left to incubate at room temperature for 1 h. After incubation, the samples were centrifuged at 10,000 g for 20 min at 4 °C. The resulting pellet was washed with 70% ethanol, dried, and finally resuspended in sterile dH2O. For PCR amplification, 50 ng of genomic DNA was used, and the PCR products were analyzed on 1% agarose gels. Primers used were: Forward primer: 5′-AGAGAGCGAGAAATCAAGCGAGTG-3′, reverse primer: 5′-CTGCCGATGTCTTTCAGACTTCAA-3′.

### RNA isolation and qRT-PCR

RNA for in vitro culture experiments was isolated at the indicated timepoints using TRIzol per the manufacturer’s instructions. RNA was resuspended in nuclease-free dH_2_O and measured using a NanoDrop. For reverse transcription reactions, 1 µg of RNA/sample was diluted to a total volume of 10 µL/sample, and generation of complementary DNA was mediated by M-MLV reverse transcriptase following the manufacturers’ instructions (ThermoFisher, cat. no. 28025021). Final samples were diluted to a volume of 200 µL prior to RT-PCR analysis. qRT-PCR was performed using FastStart Essential DNA Green Master Mix (Roche, cat. no. 06924204001) on a Roche LightCycler 96 in 96-well plates. Relative expression was measured using the 2^−DDCt^ method, and samples were normalized to *actin* expression.

**Table:** 

Gene	Forward	Reverse
*Pax7*	5′-CTGGATGAGGGCTCAGATGT-3′	5′-GGTTAGCTCCTGCCTGCTTA-3′
*Myog*	5′-TGCCCAGTGAATGCAACTCC-3′	5′-TTGGGCATGGTTTCGTCTGG-3′
*Sox4*	5′-ACAGCGACAAGATTCCGTTCA-3′	5′-CCGACTTCACCTTCTTTCGC-3′
*Sox11*	5′-CGACGACCTCATGTTCGACC-3′	5′-GACAGGGATAGGTTCCCCG-3′
*Sox12*	5′-GGAGACGGTGGTATCTGGG-3′	5′-ATCATCTCGGTAACCTCGGGG-3′
*Egr3*	5′-CCGGTGACCATGAGCAGTTT-3′	5′-TAATGGGCTACCGAGTCGCT-3′
*Lmod2*	5′-ACCTTATCCCGATTTGCTGAAG-3′	5′-ACCTTGAGCATGTCTGCAATG-3′
*Myh3*	5′-AAAAGGCCATCACTGACGC-3′	5′-CAGCTCTCTGATCCGTGTCTC-3′

### Muscle fiber isolation and culture

For ex vivo muscle fiber culture, extensor digitorum longus (EDL) was carefully dissected from each mouse and digested with 2 mg/mL of type I collagen in DMEM for 1 h at 37 °C with gentle inversions every 5 min. Individual muscles were transferred to pre-warmed DMEM to stop the digestion and triturated using a glass pipette to dissociate individual muscle fibers. Fibers were fixed immediately after digestion and dissociation (0 h) or cultured in 20% FBS, 4 ng/mL bFGF, and 1% penicillin–streptomycin in DMEM (Gibco) for 72 h at 37 °C with 5% CO_2_. For in vitro analysis of *Pax7*^CreER^ muscle fibers, fibers were cultured for 72 h in medium containing 4-hydroxytamoxifen (4-OH, 0.4 µM, Sigma) or methanol (MeOH, 1:1000). Muscle fibers were then fixed and processed as described above in 48-well plate.

### Primary myoblast isolation and culture

Primary myoblasts from *Pax7*^CreER^ or *Rosa26*^CreER^ litters were isolated using a modified protocol as described [65]. Briefly, hindlimb muscles were dissected, placed in cold 1 × PBS, washed 3 × with 1 × PBS to ensure all hair was removed, and gently cut by scraping in the direction of the muscle fiber to break up the tissue. Connective tissue and fat were removed and dissected samples were placed in 10-mL digestion buffer per mouse (700 U/mL type II collagenase, 10% horse serum, 1% penicillin–streptomycin in Ham’s F-10) and incubated at 37 °C, 100 rpm for 1 h. Samples were neutralized with wash buffer (10% horse serum, 1% penicillin–streptomycin in Ham’s F-10), centrifuged at 500 × g for 5 min, aspirated to leave 10-mL medium, and incubated with 100 U/mL type II collagenase and 1U/mL dispase II at 37 °C, 100 rpm for 30 min. Samples were run through an 18-gauge needle 10° to break up additional pieces of muscle, run through a 40-µm filter, centrifuged, supernatant aspirated and resuspended in 2-mL myoblast growth medium (20% FBS, 4 ng/mL bFGF, 1% penicillin–streptomycin in Ham’s F-10), and added to 10-cm dish/sample containing myoblast growth medium. Cells were cultured for 48 h to allow for myoblast proliferation, collected and digested with 0.025% trypsin–EDTA (Gibco), and pre-plated in 10-cm dish for 45 min to allow for fibroblasts to attached. Supernatant from each plate was collected and placed on rat-tail collagen-coated plastic 10-cm dishes, cultured for 24–48 h, and then collected and allowed to expand on non-coated plates to generate highly pure myoblasts. For proliferation, myoblasts were seeded on 24-well Matrigel-coated plates in equal concentrations and evaluated after 24 h. To induce differentiation, myoblasts were seeded in equal concentrations in 24-well Matrigel-coated plates, grown for 24 h in proliferation medium, switched to differentiation medium (2% horse serum, 1% penicillin–streptomycin in DMEM), and collected or analyzed at the indicated timepoints. For mixed cell cultures, cells collected immediately after isolation were plated on Matrigel-coated plates in equal concentrations and grown in myoblast growth medium for 24 h and then induced to differentiate via serum starvation (2% horse serum, 1% penicillin–streptomycin in DMEM) with either 4OH or MeOH. Samples + 4OH or + MeOH were compared for both control mice or the experimental. However, samples pictured and analyzed *Pax7*^CreER^;Sox11^floxed/floxed^ or *Rosa26*^CreER^;Sox11^floxed/floxed^ cultured with MeOH or 4OH. Samples were then collected for RNA isolation or immunofluorescence as described.

### Muscle fiber cross-sectional area and image analysis

For muscle fiber cross-sectional area analysis (CSA), dystrophin or laminin was used to delineate fiber boundary (as indicated). For all quantifications, images taken on 10 × objective were dragged into Fiji and analyzed using MyoSight [75]. For injured muscle, only muscle fibers with at least 1 centrally located nucleus were counted. For each pair of mice, > 3 images were analyzed spanning the muscle section to yield > 300 fibers/mouse analyzed. The frequency distribution is the average of at least 3 pairs of mice. For fiber-type and CSA analysis, images of the entire muscle section were taken, loaded into MuscleJ, and run to detected type IIA and type IIB (thus, type IIX is inferred). Pax7 and myogenin-positive cells per field of view is quantified as the average of the number of Pax7 or myogenin-positive cells over 3–4 images/mouse for minimum of 3 biological replicates.

### Quantification and statistical analysis

Samples were plotted using GraphPad Prism 8. For all samples, at least 3 pairs were used. All samples are litter, age, and gender matched. Unpaired *t*-test in Prism was used to analyze differences between genotypes.

## Results

### Identification of Sox11 expression in myogenic progenitors

To identify key regulatory factors, pathways, and mechanisms that stabilize cell identity and cell fate transitions, we first probed our previously published scRNA-seq dataset to determine genes that were uniquely enriched in the subsets of MuSCs [68]. We found that *Sox11* expression is restricted to the MuSC population in cells profiled from non-injured and injured muscles at various timepoints throughout the regenerative process (Fig. S1A). We confirmed the expression on an aggregated scRNA-seq dataset [76] and found that indeed *Sox11* expression is mostly detected in the MuSC population (Fig. 1B, C). Additional analysis of published datasets [41, 51, 77] further showed that *Sox11* expression is specifically enriched in differentiating MuSCs (Fig. 1D, Fig. S1B). Quantitative qPCR analysis confirmed an increased expression of Sox11 during primary myoblast differentiation, along with Myod and Myog (Fig. 1E). This specificity was unique to *Sox11*, as *Sox4* expression gradually declined during differentiation (Fig. 1E).

We also employed in silico scTenifoldKnk to predict perturbations in gene regulatory networks that would be caused by knockout of *Sox11* [73]. Consistent with the expression pattern of Sox11 during MuSC differentiation, the top predicted gene targets impacted by knockout of Sox11 are *Myh3*, *Lmod2*, *Mylk4*, *Myh8*, and *Mymk*, among others (Table S1). Many of these genes are developmentally expressed myosins and known regulators of newly regenerated muscle fibers [78]. Together, these preliminary analyses identified *Sox11* as a novel marker and potential regulator of MuSC differentiation.

### Age-related decline of Sox11 expression is associated with reduced chromatin contacts surrounding the Sox11 locus

**Fig. 2:**
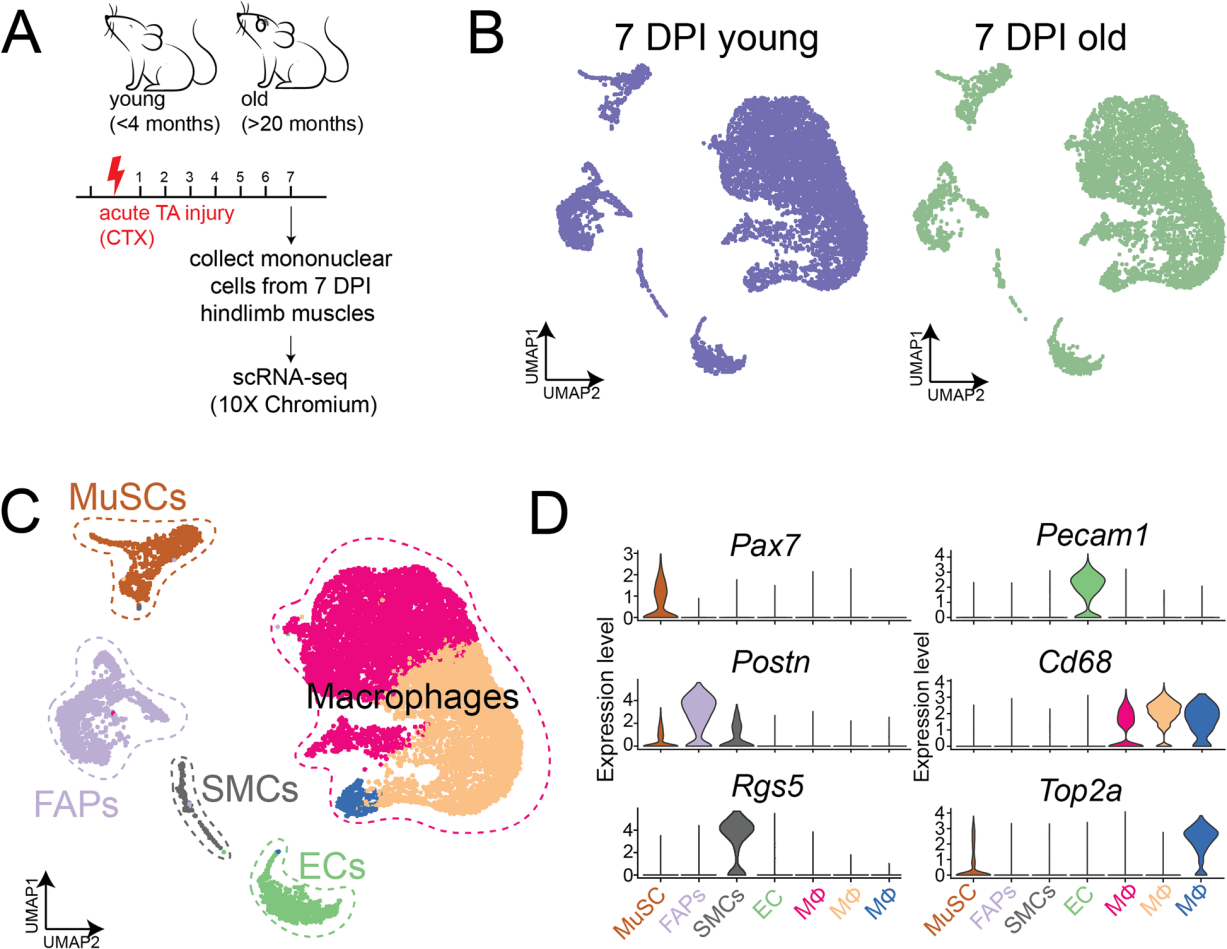
Single-cell RNA sequencing of young and old mononuclear cells from 7 DPI-injured muscle. **A** Experimental outline: mononuclear cells from hindlimb muscle from young (< 4 months old, *n* = 3) and old (> 20 months old, *n* = 3) male mice injured with CTX, collected at 7 DPI, and processed for scRNA-seq using the 10 × chromium platform. **B** UMAP of cells clustered together and separated based on young (left panel) and old (right panel) samples. **C** UMAP and clustering results of cells combined from both young and old mice. Colored based on clusters identified. **D** Violin plots for selected top marker genes used to label each cluster

Given that MuSC function declines with age [79–81], we sought to determine the cell autonomous and non-cell autonomous factors that are linked to age-related functional decline. To this end, we employed the 10 × chromium platform to garner the dynamics of transcriptional profiles of all mononuclear cells from regenerating hindlimb muscles of young (< 4 months) and old (> 22 months) male mice at 7 days post injury (DPI) (Fig. 2A). We processed both samples together and performed dimensionality reduction, clustering, and UMAP embedding of the two samples (Fig. 2B). This yielded 7874 quality cells from the young muscle samples and 6013 quality cells from the old muscles. We identified known muscle cell populations based on marker gene expression and labeled them accordingly. This included a fibro-adipogenic progenitor (FAP) population expressing *Pdgfra* and *Postn*, 3 macrophage populations expressing *Cd68* and *Mrc1*, endothelial cells (ECs) enriched for *Pecam1*, smooth muscle cells (SMCs) expressing *Rgs5*, and a muscle stem cell (MuSC) population enriched for *Pax7* (Fig. 2C, D). This recapitulated known cell populations involved in regeneration and allowed us to further probe any subtle differences in cell dynamics and gene expression that cannot be determined at the global population level.

**Fig. 3:**
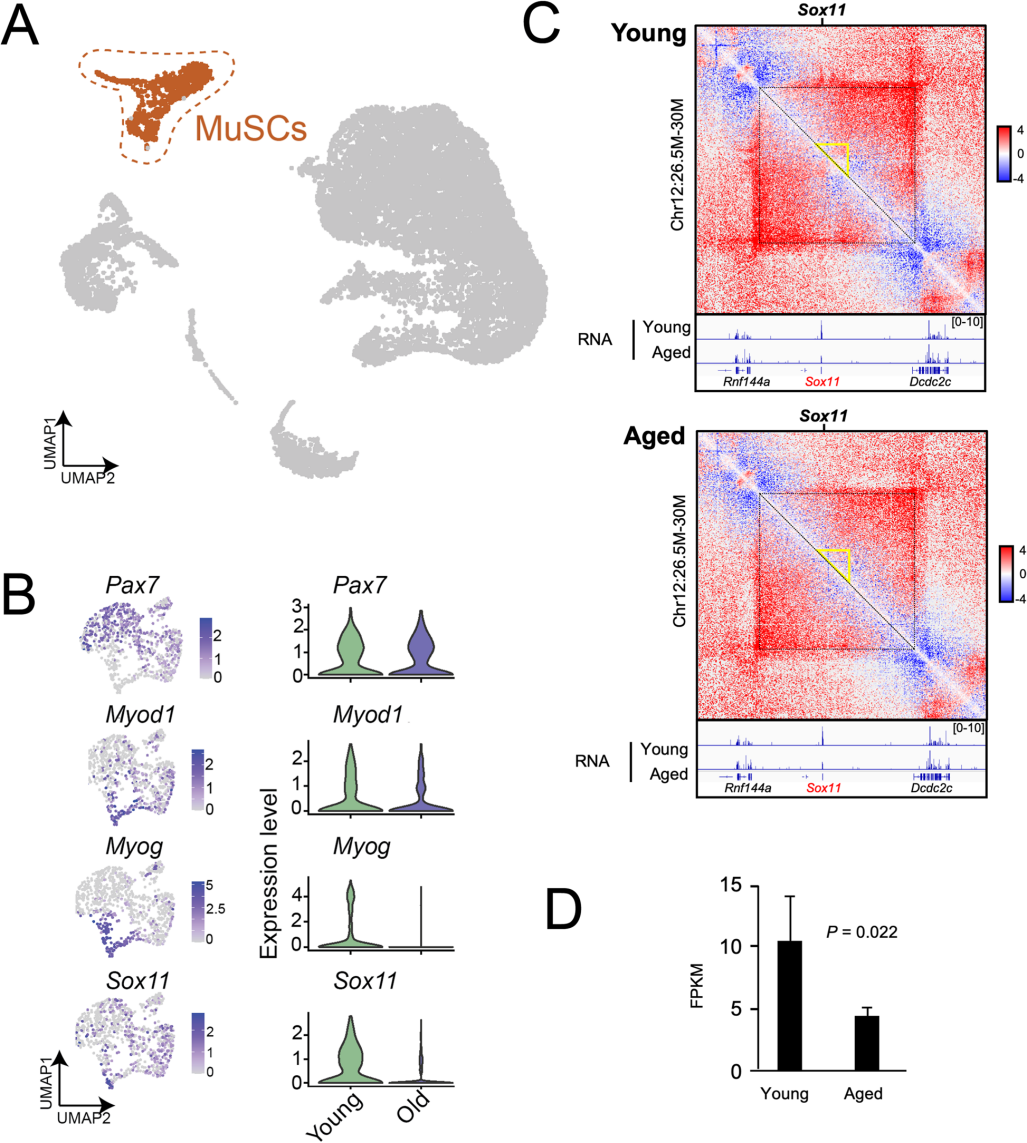
Reduced *Sox11* expression and 3D-genome alterations at *Sox11* locus in muscle stem cells with age. **A** UMAP of all cells from young and old mice, MuSCs highlighted in color. **B** Clustering and gene expression analysis on MuSCs only, highlighted in panel (A); gene expression plots (left panel) and violin plots (based on age) identifies various differentiation-related transcripts are reduced with age identifies significantly reduced *Sox11* expression in MuSCs from old mice. **C** Comparison of Hi-C contact maps (10-kb resolution) surrounding Sox11 locus between young (top) and aged (bottom) FISCs. The yellow triangle indicates the interaction region harboring Sox11 locus. Bottom, genome browser tracks showing the RNA-seq in young and aged FISCs. **D** Bar graph showing the mean FPKM values of Sox11 in young and aged FISCs. *n* = 3 for each group

To determine any subtle changes in gene expression in young compared to old MuSCs, we focused on the MuSC population by subsetting on and re-culstering the MuSC population (Fig. 3A, B). We found that while *Pax7* was relatively similar in young and old MuSCs, markers associated with differentiation (*Myod1* and *Myog*) were significantly reduced in the MuSC subset. Interestingly, *Sox11* expression was also significantly reduced. Thus, *Sox11* expression, which increases as MuSCs differentiate, may also be reduced as a consequence of the delayed kinetics associated with age-related functional decline [79].

Recent findings indicate that 3D genome reorganizes and underpins the transcriptome remodeling during SC aging [82–84]. Thus, we leveraged our recently published Hi-C datasets [82] to investigate the 3D organization around *Sox11* locus. In freshly isolated MuSCs (FISCs) from young mice, we observed substantial chromatin contacts surrounding the *Sox11* locus, which appeared reduced in the FISCs from aged MuSCs (Fig. 3C). Consistently, *Sox11* expression was significantly reduced in the bulk RNA-seq data (Fig. 3D), which is in agreement with our observations in scRNA-seq analysis. The above data indicate that the decline in Sox11 expression during MuSC aging is associated with 3D genome reorganization at *Sox11* locus.

### Sox11 is dispensable for adult muscle stem cell function and muscle regeneration

**Fig. 4:**
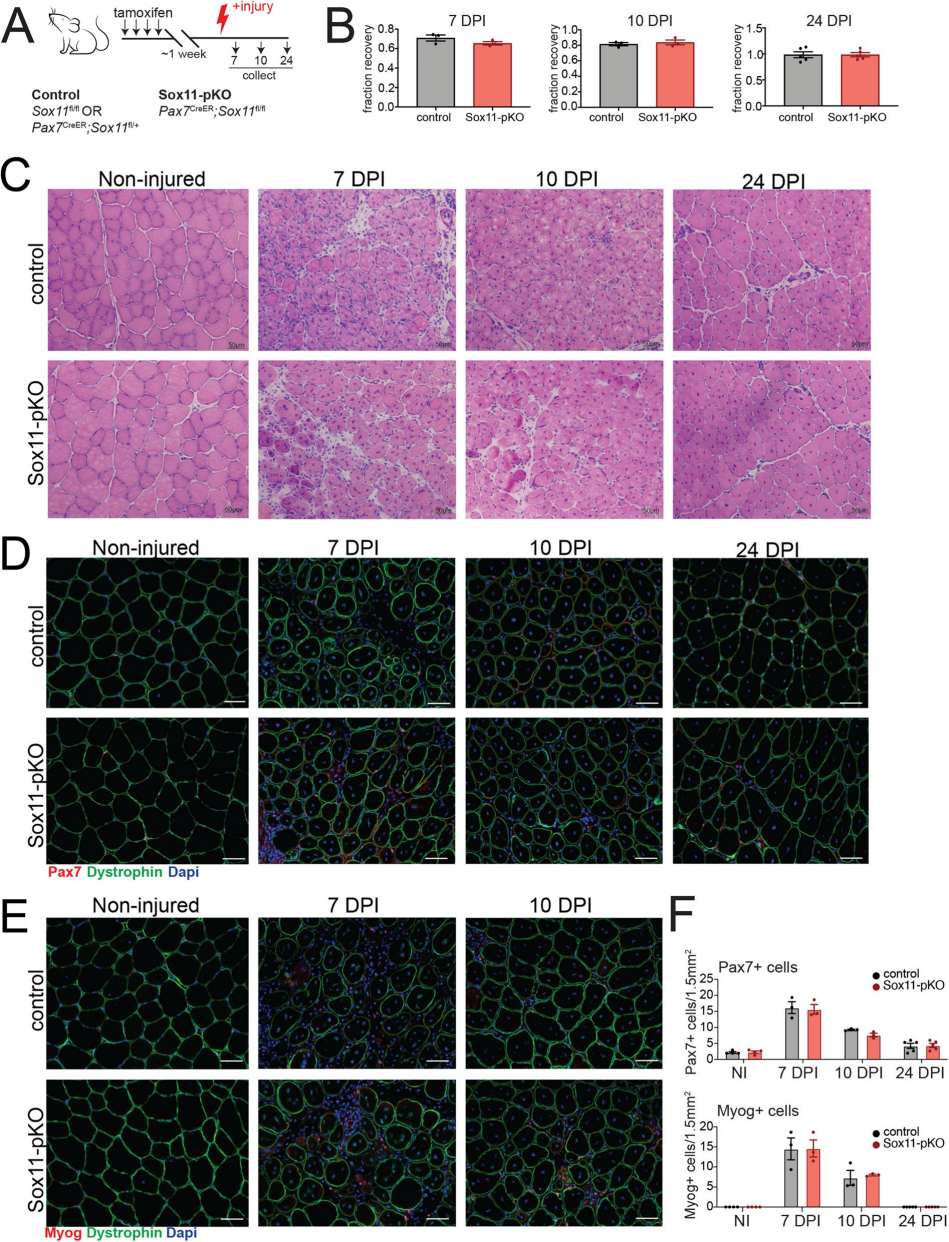
Analysis of muscle fiber area and type for Sox11-pKO mice. **A** Frequency distribution pots for CSA of TA muscle fibers from non-injured, 7, 10, and 24 DPI mice from control and Sox11-pKO mice. Measurements binned in 400 µm^2^ bins. **B** Representative immunofluorescence images to detect fiber type IIA, type IIB, dystrophin, and nuclei (DAPI) on non-injured muscles from control and Sox11-pKO mice (top panel), output from MuscleJ (bottom panel). **C** Frequency distribution pots for CSA of TA muscle fibers from non-injured muscle, separated by inferred fiber type as analyzed from MuscleJ. Measurements binned in 400 µm^2^ bins. **D** Representative immunofluorescence images to detect fiber type IIA, type IIB, dystrophin, and nuclei (DAPI) on 24 DPI muscles from control and Sox11-pKO mice (top panel), output from MuscleJ (bottom panel). **E** Frequency distribution pots for CSA of TA muscle fibers from 24 DPI muscle, separated by inferred fiber type as analyzed from MuscleJ. Measurements binned in 400 µm^2^ bins

To determine if the dynamic expression of *Sox11* during myogenesis and aging underlies a function of Sox11 protein in adult MuSC function, we generated a *Pax7*^CreER^;*Sox11*^fl/fl^-inducible mouse model to specifically delete *Sox11* in Pax7 + MuSCs and their progeny upon the administration of tamoxifen (Fig. 4A). To evaluate MuSC function upon loss of *Sox11*, we used a model of acute injury to evaluate MuSCs’ ability to activate, proliferate, and differentiate in vivo [85]. To assess the impact that loss of *Sox11* has on the ability of the MuSC pool to efficiency repair damaged fibers, we administered tamoxifen to control (*Pax7*^CreER^;*Sox11*^fl/+^ or *Sox11*^fl/fl^) and *Pax7*^CreER^;*Sox11*^fl/fl^ (Sox11-pKO), induced acute injury to the tibialis anterior (TA) muscles, and analyzed muscle regeneration at discrete timepoints throughout the regenerative process (Fig. 4A).

No significant differences in muscle mass recovery (weight) were observed between the two genotypes at 7, 10, and 24 DPI, suggesting that Sox11-pKO MuSCs are functionally competent and can repair injured muscles (Fig. 4B). Normal muscle morphology was observed in control and Sox11-pKO mice, with no clear differences in regenerated myofibers between the two genotypes at 7 and 10 DPI (Fig. 4C). By 24 DPI, both control and Sox11-pKO injured muscle appeared nearly fully repaired (Fig. 4C). Additional analysis of muscle fiber cross-sectional area (CSA) at 7, 10, and 24 DPI indicated that control and Sox11-pKO regenerated fiber area were comparable at each timepoint (Fig. S2A). While at 10 DPI, Sox11-pKO had significantly more fibers of 1200–2800 µm^2^; this modest change in size distribution was resolved by 24 DPI (Fig. S2A).

To determine if loss of *Sox11* expression alters the activation, proliferation, or differentiation status of MuSCs, we evaluated both Pax7 and myogenin expression via immunofluorescence on muscle sections from each injury timepoint (Fig. 4D, E). Both control and Sox11-pKO had comparable numbers of Pax7 + cells and Myog + cells per field of view (FOV) in non-injured and at 7, 10, and 24 DPI muscle (Fig. 4F). Specifically, both genotypes had nearly 20 Pax7 + cells/FOV at 7 DPI, which decreased to 10 and 5 by 10 and 24 DPI, respectively. The number of Myog + cells/FOV reached an average of 15 at 7 DPI for both genotypes and subsequently decreased to less than 10 by 10 DPI.

Since another Sox family member, Sox6, regulates slow-muscle fiber genes, we evaluated fiber-type distribution of non-injured and regenerated muscle at 24 DPI [54, 55]. However, fiber type and size distribution at 24 DPI were similar between control and Sox11-pKO, indicating that *Sox11* is required for muscle fiber type determination after acute TA muscle injury (Fig. S2B). Thus, Sox11 appears dispensable for MuSCs function in response to acute injury.

Multiple rounds of injury can exacerbate any subtle differences in regenerative capacity while also assessing the ability of MuSCs to self-renew [86]. To determine if *Sox11* knockout changes the proportion of self-renewing and differentiating MuSCs, we performed repetitive rounds of injury and analyzed muscle at 7 days after the 3rd injury (Fig. S3A). Sox11-pKO recovery TA weight was significantly greater than control, although gross muscle morphology and regeneration appeared comparable between control and Sox11-pKO (Fig. S3B, S3C). Interestingly, we observed significantly more Pax7 + cells per FOV in Sox11-pKO at 7DPI after the 3rd injury, which was greater than 10/FOV in Sox11-pKO compared to an average of 7.5 Pax7 + cells/FOV in control (Fig. S3C). We did not observe a significant difference in the number of Myog + cells/FOV between the two genotypes (Fig. S3C). Thus, loss of *Sox11* may slightly delay the differentiation of MuSCs which is exacerbated by multiple rounds of injury. Nonetheless, Sox11 is not absolutely required for adult MuSC function in response to acute injury or for efficient regeneration after multiple rounds of injury.

### Sox11 is expendable for muscle stem cell function in vitro

**Fig. 5:**
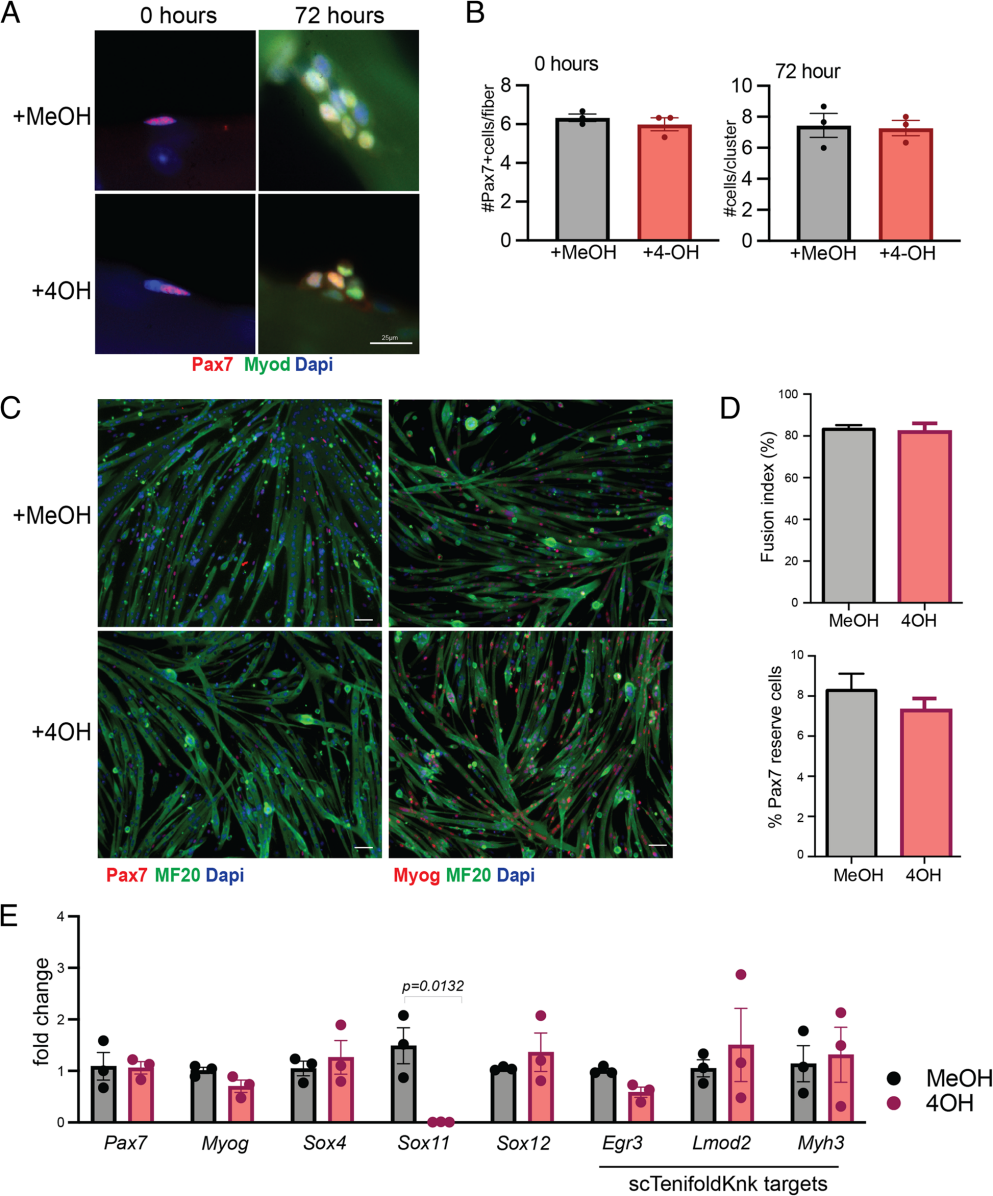
In vitro analysis of myogenic function Sox11-null myoblasts. **A** Representative immunofluorescence images to detect Pax7, Myod1, and nuclei (counterstained with dapi) of myofibers and their associated MuSCs isolated from *Pax7*^CreER^; *Sox11*^fl/fl^ EDL muscle and fixed at 0 h (left panel) or cultured for 72 h with vehicle (MeOH) or 4-OH (right panel). **B** Quantification of the number of Pax7 + cells/myofiber at 0 h (left graph) and the number of cells/clusters at 72 h in culture (right graph), related to **A**. **C** Primary myoblasts isolated from *Pax7*^CreER^; *Sox11*^fl/fl^ were cultured with MeOH (top panel) or 4-OH (bottom panel) plated at equal concentrations and induced to differentiate via serum starvation for 4 days to evaluate myogenic potential. Representative immunofluorescence images to detect Pax7, MF20, nuclei (DAPI) shown in the left panel, and Myog, MF20, and nuclei (DAPI) are shown in the right panel. **D** Quantification of the fusion indices of MeOH and 4OH-treated myoblasts, represented as the percent of nuclei fused into myotubes (top graph) and Pax7 “reserve” cells, counted as the total number of Pax7 + cells per field of view for each biological replicate (bottom graph). **E** Relative expression as measured by qRT-PCR for MuSC marker *Pax7* and differentiation marker *Myog*, members of the SoxC subfamily (*Sox4*, *Sox11*, and *Sox12*), and genes predicted to be reduced by scTenifoldKnk (*Egr3*, *Lmod2*, *Myh3*). Scale bars: 25 µm in **A**, 50 µm in **C**

The observation that Sox11-pKO mice had a greater proportion of Pax7 + cells per FOV after multiple rounds of injury (Fig. S3) prompted us to further probe if Sox11 KO influences MuSC function cell autonomously. We isolated muscle fibers and their associated MuSCs from Sox11-pKO mice and cultured them in the presence of 4-hydroxy tamoxifen (4-OH) to induce recombination of the floxed allele or MeOH (vehicle control). We fixed fibers at 0 h and 72 h in culture and used immunofluorescence to detect Pax7 and Myod (Fig. 5A). However, there was no significant differences between the MeOH- or 4OH-treated muscle fibers, with both genotypes averaging approximately 8 cells/cluster (Fig. 5B).

To further evaluate MuSC proliferation and differentiation, we isolated primary myoblasts from Sox11-pKO mice and cultured them in the presence of 4OH or MeOH. We confirmed recombination of the floxed allele upon the additional of 4OH in culture (Fig. S4A) and subsequently evaluated proliferation and differentiation. Sox11-pKO myoblasts treated with MeOH or 4-OH had comparable numbers of Pax7 + /Ki67 + cells and comparable numbers of Myog + cells (Fig. S4B) suggesting no overt proliferative and differentiation defects. Given that *Sox11* was enriched during differentiation, we induced differentiation through serum starvation of Sox11-pKO myoblasts treated with 4OH or MeOH (vehicle control). Immunofluorescence on differentiated cells to detect Pax7 or Myog and MF20 suggested control (+ MeOH), and Sox11-null (+ 4-OH) myoblasts differentiated similarly (Fig. 5C). Control and Sox11-null myoblast fusion indices both reached 80%, and the number of “reserve” Pax7 + cells, which may represent self-renewal capacity of myoblasts [87], was comparable with MeOH treated reaching near 8% and 4OH treated reaching near 7% Pax7 reserve cells (Fig. 5D). Gene expression analysis on RNA isolated from differentiated myoblasts confirmed that *Sox11* expression was ablated, while *Pax7* and *Myog* were unperturbed in the knockout cells (Fig. 5E). The expression of the other SoxC genes, *Sox4* and *Sox12*, was unchanged between the two conditions, excluding the possibility that a compensatory increase in expression of other Sox members may have accounted for the lack of phenotype (Fig. 5E). We also evaluated the expression of scTenifoldKnk Sox11-KO genes that were predicted to be dysregulated upon Sox11 knockout and found no significant differences in their expression upon loss of *Sox11* (Fig. 5E). In conclusion, *Sox11* is not required for MuSC self-renewal or differentiation in vitro.

### Loss of Sox11 in muscle progenitors minimally impacts muscle development

Although we determined that *Sox11* is unnecessary for adult MuSC function, many of the Sox family of transcription factors are highly expressed during development and are required for proliferation and survival as well as differentiation of various embryonic cell types [35, 36]. We therefore evaluated the requirement of *Sox11* for muscle progenitor function during development by generating *Myod1*^Cre^; *Sox11*^fl/fl^ mice to specifically delete Sox11 in Myod1 + muscle progenitors and their progeny. Control (*Myod1*^Cre^;*Sox11*^f*l*/+^, *Sox11*^f*l*/fl^, or *Sox11*^f*l*/+^) and *Myod1*^Cre^;*Sox11*^f*l*/fl^ (Sox11-mKO) mice were born in Mendelian ratios and had comparable body weights (Fig. S5A). There were no observable differences in muscle morphology or CSA by 3 months of age between control and Sox11-mKO mice (Fig. S5B, C). Therefore, although Sox11 is important for tissue specification and organogenesis, it does not appear overtly necessary for normal muscle development. To further probe the impact that loss of *Sox11* in muscle progenitors has on MuSC function, we induced acute injury via intramuscular injection of CTX and analyzed samples at 5.5 DPI (Fig. S5A). Control and Sox11-mKO had similar TA recovery weights and no obvious differences in muscle morphology and regeneration 5 DPI (Fig. S5B, S5C). Immunofluorescence on muscle sections to detect Pax7 or Myog and Laminin supported the lack of observable differences between control and Sox11-mKO, with control and Sox11-mKO having an average of 35 Pax7 + cells/FOV and an average of approximately 20 Myog + cells/FOV for both genotypes (Fig. S5C). Thus, *Sox11* is dispensable for the establishment and function of the MuSC pool.

### Loss of Sox11 does not accelerate age-related functional decline

**Fig. 6:**
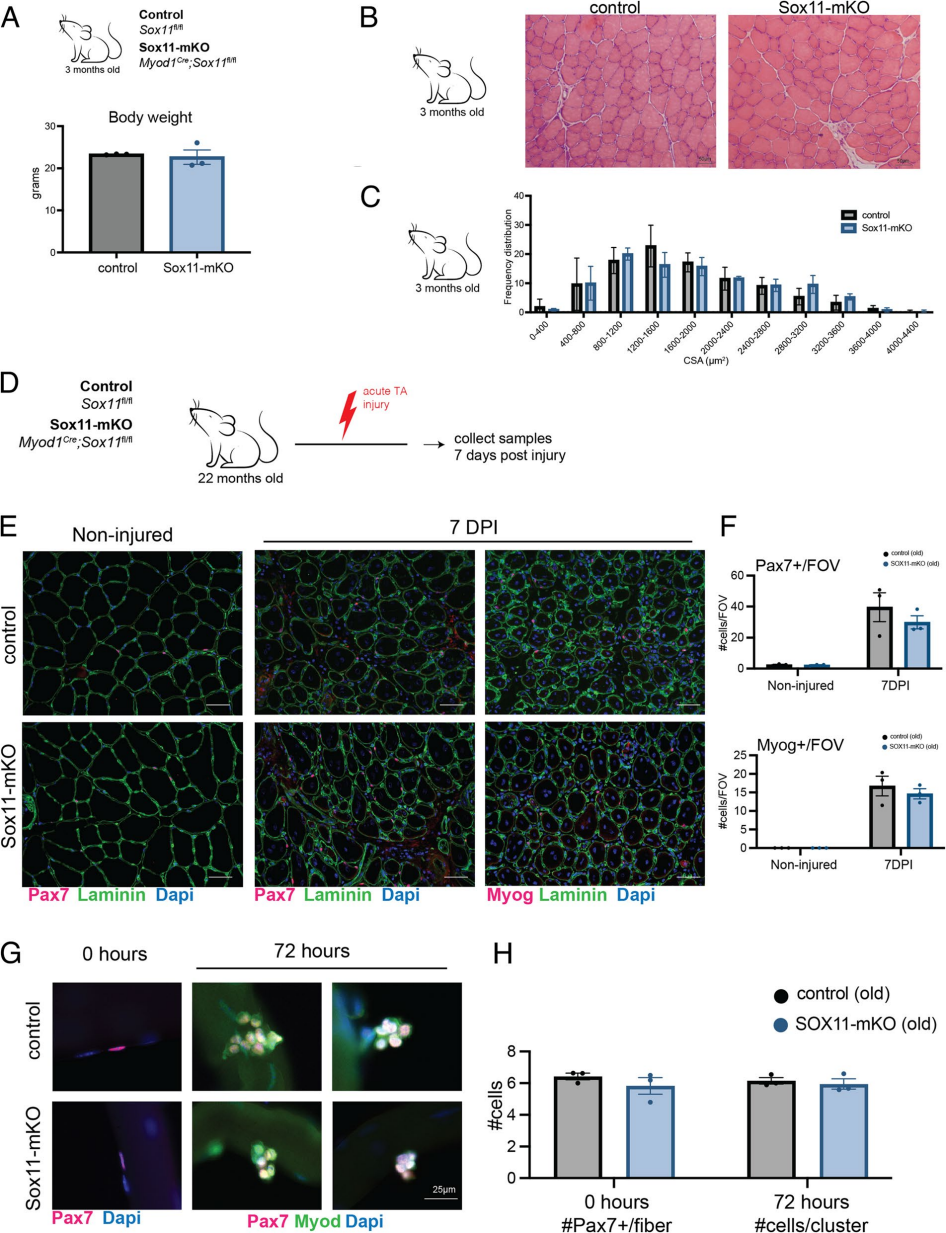
Requirement of Sox11 in muscle development and aging. **A** Body weight of control and Sox11-mKO mice in grams. **B** H&E staining and representative images of 10-µm TA muscle cross-sections from control (top panel) and Sox11-mKO (bottom panel) non-injured samples, scale bars: 50 µm. **C** TA muscle fiber CSA for non-injured control and Sox11-mKO mice, related to **B**. **D** Experimental outline to evaluate the impact loss of Sox11 has on regeneration of aged (> 20-month-old) mice. The TA muscle of control and Sox11-mKO mice were injured via intramuscular injection of CTX and collected at 7 DPI to evaluate regeneration. **E** Representative images of immunofluorescence on TA muscle sections to detect Pax7 or Myog, dystrophin, and nuclei (counterstained with DAPI) from control (top panel) and Sox11-mKO (bottom panel) old mice at 0 (non-injured) and 7 DPI, scale bars: 50 µm. **F** Quantification of the number of Pax7 + cells/FOV (top graph) and Myog + cells/FOV (bottom graph) for control and Sox11-pKO mice. **G** Ex vivo culture of muscle fibers isolated from EDL of old control (top panel) and Sox11-mKO (bottom) mice, fixed at 0 h (left panel) or after 72 h in culture (right panel) and stained to detect Pax7, Myod, and nuclei (DAPI), scale bar 25 µm. **H** Quantification of the number of Pax7 + cells/myofiber at 0 h (left graph) and the number of cells/clusters at 72 h in culture (right graph), related to **G**

Since we identified *Sox11* expression to be reduced in old MuSCs from 7 DPI and found age-related changes in chromatin conformation (Fig. 3), we sought to evaluate whether loss of *Sox11* exacerbates any age-related regenerative function [34, 88]. We therefore injured the TA muscles of old (~ 22 months old) control and Sox11-mKO mice and analyzed samples at 7 DPI (Fig. 6D). Both control and Sox11-mKO mice regenerated efficiently, as evidenced by immunofluorescence to detect Pax7 or Myog and laminin which were indistinguishable (Fig. 6E). Quantification of the number Pax7 + or Myog + cells per FOV indicated age did not lead to any obvious alterations in MuSC’s ability to repair damaged fibers, both having an average of approximately 35 Pax7 + cells/FOV and 15 Myog + cells/FOV at 7 DPI (Fig. 6F). While measurement of muscle fiber CSA suggested that aged Sox11-mKO had significantly fewer fibers in the range of 2000–2400 µm^2^, regenerated fiber CSA was similar between control and Sox11-mKO (Fig. S5D). Ex vivo fiber and MuSC culture results were consistent with in vivo results, with no significant differences observed in Pax7 + cells per myofiber or cluster size (Fig. 6G, H). Therefore, although we identified reduced *Sox11* expression in MuSCs from aged mice, loss of *Sox11* does not appear to accelerate any age-related regenerative capacity of MuSCs.

### Sox11 is globally dispensable for muscle repair after acute injury

A recent study specifically performed scRNA-seq on nuclei isolated from muscle spindles and found *Sox11* expression to be unique to the sensory bag fibers [89]. Furthermore, Sox11 is required for sensory neuron regeneration and thus may more broadly play a role in regeneration [41, 51]. To understand if Sox11 is globally required for adult muscle regeneration, we crossed *Rosa26*^CreER^ mice with *Sox11*^fl/fl^ mice to delete *Sox11* in all cell types upon the administration of tamoxifen. We administered tamoxifen to control (*Rosa26*^CreER^;*Sox11*^fl/+^, *Sox11*^fl/fl^ or *Sox11*^fl/fl^) and *Rosa26*^CreER^;*Sox11*^fl/fl^ mice (Sox11-KO) and confirmed the efficiency of recombination of the *Sox11* floxed allele in whole muscle and MuSCs purified by fluorescence-activated cell sorting (Fig. S6A). We then induced acute muscle injury with BaCl_2_ and analyzed regeneration at 5, 7, 10, and 24 DPI (Fig. S6B). Control and Sox11-KO recovery TA weights were similar, suggesting broadly efficient muscle regeneration between the two genotypes (Fig. S6C). Cross-section analysis of non-injured and injured TA muscles revealed similar morphological characteristics between control and Sox11-KO at each regeneration timepoint (Fig. S6D). Additional analysis of TA muscle sections using immunofluorescence for dystrophin, Pax7, and myogenin further supported the relatively similar regenerative capacity of control and Sox11-KO (Fig. S6E, F). Quantification of the number of Pax7 + and Myog + cells per FOV indicated control, and Sox11-KO mice were indistinguishable across the timepoints assayed (Fig. S6G). We therefore concluded that Sox11 is globally dispensable for adult muscle regeneration. Thus, while Sox11 is required for a variety of developmental processes, its role in muscle function and repair response to acute injury is limited.

## Discussion

The SOX family TFs play diverse roles in regulation of cell identity, self-renewal, and differentiation through their modulation of various transcriptional programs [90]. For example, during skeletogenesis, Sox11 was found to stabilize nuclear β-catenin, thereby promoting canonical WNT signaling to secure cell fate and shown to specifically regulate WNT-related pathway genes required for mesenchyme specification and organogenesis [59, 91]. During muscle development, WNT ligands are secreted from the neural tube to promote myogenesis of the adjacent somite [31, 92]. In resting adult muscle, the WNT signaling pathway is not active but is activated in response to injury and in vitro during differentiation, and increased WNT signaling impairs the regenerative potential of MuSCs [33, 34, 80, 93, 94]. Interestingly, β-catenin-dependent WNT signaling opposes Notch signaling to promote MuSC differentiation, and Sox11 was also shown to regulate the expression of Notch-related pathway genes [46, 48, 49, 95]. We identified *Sox11* expression to be enriched in differentiating MuSCs and reduced with age, consistent with age-dependent changes in chromatin conformation at the *Sox11* locus. We therefore hypothesized that Sox11 may play a role in the transcriptional regulation of MuSC function and fate decisions.

To this end, we employed a handful of genetic models to evaluate the requirement for Sox11 in muscle development and regeneration. To evaluate the requirement for Sox11 in the adult muscle stem cell pool, we used a tamoxifen-inducible MuSC-specific Pax7^CreER^ to delete *Sox11* in MuSCs. We sampled muscle at 7, 10, and 24 DPI to garner a complete picture of the regenerative process. The results indicated that control and Sox11-pKO exhibit comparable rates of regeneration. These data were corroborated by ex vivo myofiber culture and in vitro differentiation of myoblasts. While Sox11-pKO muscles had an increased number of Pax7 + MuSCs at 7 days after a 3rd muscle injury, muscle regeneration was relatively normal, with no other overt defects observed. Thus, loss of Sox11 does not impact the ability of MuSCs to activate, proliferate, and differentiate in response to a single or multiple rounds of injury.

Since the Sox family of TFs are critically known for their development roles, we deleted Sox11 in muscle progenitors (driven by Myod1^Cre^) but found that muscle developed normally and maintained regenerative capacity into adulthood. As we identified age-related reduced *Sox11* expression and changes in chromatin conformation at the *Sox11* locus, we evaluated whether loss of *Sox11* impacts regenerative capacity with age. Surprisingly, we did not observe overt differences in regenerative capacity with age either. To outline the potential non-cell-autonomous requirement for Sox11 in adult skeletal muscle regeneration, we deleted Sox11 in all adult cells (driven by Rosa26^CreER^) and induced acute muscle injury. However, assessment of the muscle regeneration after acute injury indicated that Sox11 is globally dispensable for muscle regeneration.

The lack of phenotypes after Sox11 deletion driven by three different Cre(ER) lines is surprising but can be explained by several possibilities. First, the SoxC group (Sox4/11/12) may have overlapping functions in MuSCs. Although *Sox11* is highly enriched in MuSCs, its level is much lower than that of Sox4, which is broadly expressed in many cells. In this scenario, the loss of Sox11 may be compensated by Sox4. Future studies to knockout both *Sox4* and *Sox11* would address this possibility. Second, it is possible, though unlikely, that the Sox11 deletion was not efficient in all the models used in this study. Due to the lack of commercially available and highly specific Sox11 antibodies, we cannot exclude the possibility that low levels of Sox11 protein are present in the knock out lines. We tried to address this issue by genomic DNA recombination analysis (which measures truncated *Sox11* alleles). We presented data showing recombination of the floxed alleles in MuSCs and whole muscle of Rosa26^CreER^/Sox11^flox/flox^ mice upon tamoxifen administration as well as in primary myoblasts isolated from Pax7^CreER^/Sox11^flox/flox^ mice cultured in the presence of 4-OH. While this indicated that recombination is highly efficient, we can not definitively state that recombination is 100%. Likewise, the efficiency of Myod1^Cre^-driven Sox11 DNA recombination was not evaluated due to constraints on breeding. The lack of antibody validation of Sox11 protein levels and DNA recombination assays on all three lines used represent a limitation of the current study.

Although our data indicate that Sox11 is not required for normal MuSC activation, proliferation, and differentiation in order to repair muscle injury in response to acute injury, it remains to be determined if Sox11 plays a role under other conditions, such as nerve crush injury. Nonetheless, our data provide the community with knowledge about the unique stage-specific expression yet dispensable role of Sox11 in muscle development and acute muscle injury repair.

## Conclusions

We used scRNA-seq and 3D chromatin conformation assays to demonstrate unique enrichment of Sox11 expression in MuSCs and its stage-/age-dependent expression dynamics, associated with changes in three-dimensional genome organization at the *Sox11* gene locus. Through a series of genetic assays using conditional knockout models, we found that Sox11 expression in myogenic and non-myogenic cells is dispensable for normal muscle development, MuSC regenerative function in response to injury in adulthood, and age-related muscle maintenance and regeneration in adulthood. Further studies to determine whether other SOX TFs can compensate for Sox11 may lend further insight into a potential regulatory role of Sox11 in myogenesis.

### Supplementary Information

**Additional file 1:** **Table S1.** *scTenifoldKnk* prediction of top ranked genes dysregulated by Sox11 knokcout in muscle satellite cells*. ***Fig. S1.** Sox11 expression enriched in differentiating MuSCs. A. Left: UMAP of previously published scRNA-seq dataset on mononuclear cells from non-injured, 0.5, 2, 3.5, 5, 10, and 21 DPI aggregated together and labeled based on their broad cell type (right). Right: Violin plots to how the expression of *Pax7* and *Myod1*, which are specific to the MuSC subset, and *Sox11 *whose expression is also specific to MuSCs. B. Monocle pseudotime trajectory analysis of MuSCs and gene expression plots on pseudotime of *Pax7*, *Myog* and *Sox11* from 5 DPI MuSCs shown in Fig. 1D. Related to Figure 1. **Fig. S2.** Analysis of muscle fiber area and type for Sox11-pKO mice. A. Frequency distribution pots for CSA of TA muscle fibers from non-injured, 7, 10, and 24 DPI mice from control and Sox11-pKO mice. Measurements binned in 400 um^2^ bins. B. Representative immunofluorescence images to detect fiber Type IIA, Type IIB, Dystrophin and nuclei (DAPI) on non-injured muscles from control and Sox11-pKO mice (top panel), output from MuscleJ (bottom panel). C. Frequency distribution pots for CSA of TA muscle fibers from non-injured muscle, separated by inferred fiber type as analyzed from MuscleJ. Measurements binned in 400 um^2^ bins. D. Representative immunofluorescence images to detect fiber Type IIA, Type IIB, Dystrophin and nuclei (DAPI) on 24 DPI muscles from control and Sox11-pKO mice (top panel), output from MuscleJ (bottom panel). E. Frequency distribution pots for CSA of TA muscle fibers from 24 DPI muscle, separated by inferred fiber type as analyzed from MuscleJ. Measurements binned in 400 um^2^ bins. Related to Fig. 4. **Fig. S3.** Requirement of Sox11 for regenerative capacity of muscle stem cells after multiple rounds of injury. A. Experimental outline to evaluate the requirement of Sox11 in MuSCs. Control and Sox11-pKO mice were injected with tamoxifen to induce recombination of the floxed allele. Subsequent MuSC function via muscle regeneration was evaluated using acute injury of the TA muscle with CTX 3 times (21 days between injury) and collected at 7 days post the 3^rd^ injury. B. Recovery TA weight, measured as the ratio of injured/non-injured contralateral muscle weight for 3X injured muscle. C. Representative images of immunofluorescence on TA muscle sections to detect Pax7 or Myog, Dystrophin, and nuclei (counterstained with DAPI) from control (top panel) and Sox11-pKO (bottom panel) mice at 0 (non-injured), 7 days post 3^rd^ injury D. Quantification of the number of Pax7+ cells/FOV and Myog+ cells/FOV for control and Sox11-pKO mice. Scale bars: 50 µm. *p-value = 0.01. Related to Figure 4. **Fig. S4.** In vitro analysis of Sox11-null myoblasts. A. Genomic DNA was isolated from Pax7CreER+ myoblasts homozygous for the floxed allele (fl/fl) or wild-type at the Sox11 locus +/+ either cultured in the presence of methanol (vehicle control) or 4-hydroxy tamoxifen (4OH). B. Primary myoblasts isolated from Sox11-pKO hindlimb muscle were cultured in the presence of MeOH (vehicle control) or 4-OH for 48 hours. Cells were seeded at equal concentrations, allowed to proliferate for 24 hours, fixed, and immunofluorescence was used to detect Ki67, Pax7, and nuclei (DAPI) (right panel) or Myog and nuclei (DAPI) (left panel). Bar graphs represent quantification of the number of Pax7+/Ki67+ cells per FOV or Myog+ cells per FOV. Related to Figure 5. **Fig. S5.** Loss of Sox11 in muscle progenitors in adult and old mice. A. Experimental outline to evaluate the requirement of Sox11 for muscle regeneration. Intramuscular TA injury was induced with CTX and samples were collected at 5 DPI. B. Recovery TA weight, measured as the ratio of injured/non-injured contralateral muscle weight for 5 DPI muscle. C. Representative images of immunofluorescence on TA muscle sections to detect Pax7 or Myog, Laminin and nuclei (counterstained with DAPI) from control (top panel) and Sox11-mKO (bottom panel) mice at 5 DPI and quantifications of the number of Pax7+ cells/FOV and Myog+ cells/FOV for control and Sox11-pKO mice based on 100X magnification FOV. Scale bars: 50 µm. Related to Figure 5. **Fig. S6.** Impact of global loss of Sox11 on muscle stem cell function and regeneration.  A. Genomic DNA was isolated from tibialis anterior (TA) muscle after 5 consecutive days of tamoxifen administration and subject to PCR to confirm recombination of the floxed Sox11 allele in whole muscle. Similarly, MuSCs were purified by fluorescence activated cell sorting as the CD31^-^/CD45^-^/SCA1^-^/VCAM1^+^ fraction, genomic DNA was isolated and recombination of the floxed allele evaluated by PCR to successfully confirm recombination of the floxed allele in MuSCs. B. Schematic of tamoxifen injection, muscle injury with BaCl_2_ and the respective post-injury timepoints for muscle analysis. C. Recovery TA weight for control and Sox11-pKO mice, a measured by the ratio of injured: non-injured TA muscle. D. Representative images of H&E staining from cross-sections of non-injured, 7, 10 and 24 DPI TA muscle from control (top) and Sox11-KO (bottom). E. Representative immunofluorescence images of cross-sections from control (top) or Sox11-KO (bottom) stained with antibodies to detect Pax7, Dystrophin and nuclei (DAPI), of non-injured, 5, 7, 10 and 24 DPI muscle. F. Representative immunofluorescence images of cross-sections from control (top) or Sox11-KO (bottom) stained with antibodies to detect Myog, Dystrophin and nuclei (DAPI), of non-injured, 5, 7, and 10 DPI muscle. F. Quantification of the number of Pax7+ cells/FOV (top graph) and Myog+ cells/FOV (bottom graph) for control and Sox11-KO mice based on 200X FOV. Scale bars: 50 µm.

## Data Availability

scRNA-seq data generated in this study can be found under the GEO accession number GSE226907. Previously published and used scRNA-seq data can be found under the accession numbers GSE150366 (scRNA-seq of MuSCs from non-injured, 5 and 10 DPI muscle) and GSE162172 (aggregated scRNA-seq data from skeletal muscle). Previously published in situ Hi-C and bulk RNA-seq data of young and aged MuSCs can be found under the accession numbers GSE189838 and GSE189839, respectively.

## Abbreviations

4OH: 4-Hydroxytamoxifen
BaCl_2_: Barium chloride
CSA: Cross-sectional area
CTCF: Transcriptional repressor CCCTC-binding factor
CTX: Cardiotoxin
DPI: Days post injury
ECs: Endothelial cells
FAPs: Fibro-adipogenic progenitors
FISCs: Freshly isolated muscle stem cells
FOV: Field of view
H&E: Hematoxylin & eosin
MeOH: Methanol
MRFs: Myogenic regulatory factors
MuSCs: Muscle stem cells
NICD: Notch intracellular domain
scRNA-seq: Single-cell RNA-sequencing
SMCs: Smooth muscle cells
Sox11-KO: *Rosa26*^CreER^;*Sox11*^fl/fl^ (+ tamoxifen *Rosa26*^CreER^;*Sox11*^−/−^)
Sox11-mKO: *Myod1*^Cre^;*Sox11*^fl/fl^ (in myogenic progenitors *Myod1*^Cre^;*Sox11*^−/−^)
Sox11-pKO: *Pax7*^CreER^;*Sox11*^fl/fl^ (+ tamoxifen in MuSCs *Pax7*^CreER^;*Sox11*^−/−^)
TA: Tibialis anterior
TAD: Topologically associating domain
TFs: Transcription factors
UMAP: Uniform manifold approximation
